# Examining the Role of Third Sector Organization Volunteers in Facilitating Hospital-to-Home Transitions for Older Adults – a Collective Case Study

**DOI:** 10.5334/ijic.7670

**Published:** 2024-02-29

**Authors:** Michelle L. A. Nelson, Marianne Saragosa, Hardeep Singh, Juliana Yi

**Affiliations:** 1 Lunenfeld-Tanenbaum Research Institute, Sinai Health System, CA; 2 Institute of Health Policy, Management, and Evaluation, Dalla Lana School of Public Health, University of Toronto, Toronto, Ontario, CA; 3 Department of Occupational Science & Occupational Therapy, Temerty Faculty of Medicine, University of Toronto, Toronto, Ontario, CA; 4 Rehabilitation Sciences Institute, Temerty Faculty of Medicine, University of Toronto, Toronto, CA; 5 KITE-Toronto Rehabilitation Institute, University Health Network, Toronto, CA; 6 Clinical Institutes and Quality Programs, Ontario Health, CA

**Keywords:** Transitions, third sector, case study, intermediate care, hospital discharge, person centred care

## Abstract

**Introduction:**

With increasing attention to models of transitional support delivered through multisectoral approaches, third-sector organizations (TSOs) have supported community reintegration and independent living post-hospitalization. This study aimed to identify the core elements of these types of programs, the facilitators, and barriers to service implementation and to understand the perspectives of providers and recipients of their experiences with the programs.

**Methods and Analysis:**

A collective case study collected data from two UK-based ‘Home from Hospital’ programs. An inductive thematic analysis generated rich descriptions of each program, and analytical activities generated insights across the cases.

**Results:**

Programs provided a range of personalized support for older adults and addressed many post-discharge needs, including well-being assessments, support for instrumental activities of daily living, psychosocial support, and other individualized services directed by the needs and preferences of the service user. Results suggest that these programs can act as a ‘safety net’ and promote independent living. Skilled volunteers can positively impact older adults’ experience returning home.

**Conclusions:**

When the programs under study are considered in tandem with existing evidence, it facilitates a discussion of how TSO services could be made available more widely to support older adults in their transition experiences.

## Background

The post-hospitalization period represents a vulnerable period for older adults, placing these patients at high risk for adverse health outcomes [1]. Deconditioning, or “hospitalization-associated disability,” occurs in 30% of older adults, with 16% requiring rehospitalization within 30 days [2], and rising mortality rates follow inpatient stays [3]. Some of the harmful consequences of hospitalization [4] contribute to unexpected challenges with mobility and self-care [5]. Particularly for older adults, difficulties in daily management and processing of information and an insufficient social support network exacerbate the hospital-to-home transition period [6].

Transitional care models exist to assist patients in transitioning from a hospital to an outpatient setting [7]. Transitional care is a set of actions designed to ensure the coordination and continuity of healthcare as individuals transfer between different locations or levels of care within the same location [8]. A substantial body of evidence underscores the effectiveness of hospital-based transitional care models on readmission and mortality rates, and quality of life in disease-specific populations (i.e., heart failure, stroke, chronic obstructive pulmonary disease, hip fracture) [9,10,11,12]. However, there are gaps in the care transition intervention evidence, notably around interventions focused specifically on older adults and their family caregivers as well as exploring interventions examining the value of unpaid social and community-based professional networks [13].

There has been increasing attention to models of transitional aftercare delivered through multisectoral approaches [14], defined as the deliberate collaboration among various stakeholder groups and sectors, including public (e.g., hospitals and health authorities), private (e.g., for-profit organizations), and in some countries, the third sector (e.g., voluntary organizations) [15,16]. Third-sector organizations (TSOs) are bridge the gap between the healthcare sector and the community in various ways, including providing healthcare services to underserved and marginalized populations, offering non-medical activities for to support improved well-being, and delivering health education and promotion programs [17,18]. As providers of these services to support independent living post-hospitalization services [19], TSOs are instrumental to patients’ network of care and support.

The term ‘third sector’ describes organizations that belong neither to the public (e.g., the government) nor the private (e.g., for-profit private organizations) sectors, instead, TSOs include non-profits, think tanks, registered charities, and other organizations such as self-help groups, and social enterprises, distinct from public and private sectors [20]. A central tenant for health-based professionalized TSOs is the delivery of services by a professionalized workforce of paid staff and highly trained volunteers [21]. Collaboration and partnerships between healthcare organizations and TSOs may reduce demands on health systems; engaging multiple sectors could be an opportunity to optimize existing expertise and resources to support the shared goal of improving health outcomes [22].

Volunteers contribute to inpatient care services [23,24,25] and positively impact patient-reported health outcomes [26,27,28]. Despite the historical engagement of TSOs in providing community-based support and services, research on the delivery of transitional care interventions has predominantly focused on the role of nurses, health disciplines, and other formal health care providers [29,30,31]. Limited evidence of volunteer-led care transition programs has demonstrated promising results on improved self-management behaviours and physical functioning, decreased social isolation and reduced health service utilization [26,27,32]. Therefore, despite the historical engagement of TSOs in providing community-based support and services, research on the delivery of transitional care interventions has predominantly focused on the role of nurses, health disciplines, and other formal health care providers [29,30,31]. Specifically, the role, contributions, and value of TSOs in delivering transitional care services have received less attention and need to be clearly and consistently defined [33]. Indeed, for the past decade, calls have been made for a better understanding of the role, contributions, and impact of third-sector engagement on the delivery of care [34,35,36]. This understanding could push for a more robust integration between health and social care services. Therefore, this study aimed to examine service delivery by third-sector organizations. Recognizing the potential value of TSOs and volunteers in improving client experience and outcomes and recent trends in advocating for health and social partnerships, this paper synthesizes evidence from two cases of volunteer-provided transition support programs for older people post-hospital discharge.

### Research Purpose

To identify the design elements of volunteer-supported transitions from hospital to home, programs, the facilitators and barriers to service implementation, and the perspectives of providers and recipients regarding these services to inform the development and evaluation of other such initiatives.

## Methods

### Study Design

A collective case study was conducted with research ethics approval from Sinai Health System REB [37] allowing for the exploration of a phenomenon of interest across multiple real-world settings and assessing the cases’ similarities and differences.

### Recruitment of Cases

We undertook a purposive sampling strategy comprised of a focused literature review (academic and grey literature) combined with a Google search. We consulted experts in the fields of voluntary sector engagement in healthcare and hospital transitions for the names of possible cases [38]. Through these strategies, we identified nine volunteer-provided transition support programs that met the following criteria: i) programs were delivered by TSOs, engaging volunteers as the primary point of support for clients; ii) programs were focused on returning to independent living post-hospitalization; iii) they provided services to older adults; and iv) were an established model of service (i.e., not a pilot/demonstration project or a part of a research study). We did not specify patient populations for inclusion or exclusion beyond serving older adults, as the case study focused on the program components, facilitators and barriers to implementation and participants’ perspectives regarding the service.

This paper reports on the insights gained from two UK-based TSOs ‘Home from Hospital’ programs. The included programs had similar missions and objectives: to provide support and services at different points during a person’s transition between hospital and home and engage volunteers as the primary ‘workforce’ to support individuals and formalize the volunteers’ intake mechanisms and training processes. The programs had between 50 and 70 volunteers providing services. Programmatic details can be found in Table 1. In addition to meeting inclusion criteria, the recruited sites allowed sufficient research team access to individuals and organizational documents [39].

**Table 1:** Study Sample.


PROGRAM	*UK PROGRAM 1: HOME FROM HOSPITAL SERVICE*	*UK PROGRAM 2: HOME FROM HOSPITAL SERVICE*

Number of Participants	4 Staff 9 Volunteers 4 Clients	3 Staff 6 Volunteers 4 Clients

Participants per Focus Group or Interviews	2 Focus Groups (1 with all staff, 2 with volunteers) 4 Interviews	2 Focus Groups (1 with staff, 2 with volunteers) 4 Interviews


### Participant Recruitment within Sites

Organizational leaders from agreeable sites received a study introduction package that could be shared with potential program leaders, volunteer coordinators, volunteers, and clients. Interested individuals were asked to contact the study team to receive more information, including the consent forms. Upon obtaining written informed consent, the research team members scheduled focus groups and interviews, attending each study site to collect data (see Table 1 for details regarding study samples from each case).

### Data collection

A multi-method qualitative data collection strategy (i.e., interviews, focus groups, document analysis) was applied to collect perspectives of the program or service by the administration, volunteers, and clients and the successes and challenges they experienced related to the program or service. Interviews and focus groups were recorded, transcribed, and de-identified. Program documents were also collected by retrieving public information about the programs and administrative documents provided by program staff. These documents included: information about programs on websites, forms for recruiting volunteers, lists of tasks volunteers do, risk assessment forms, discharge and referral forms, and program evaluation documents.

### Data analysis

Data analysis and collection occurred concurrently while the researchers created case summaries and wrote analytical memos using the transcripts. An inductive thematic analysis was applied by identifying, analyzing and reporting patterns (themes) within the interview and focus group data [40]. As we employed an inductive analysis using an in vivo approach, we identified codes and categories used for analyses directly from the language used by participants during data collection [41]. These codes were applied to all the interviews and focus group transcripts to generate a rich and detailed account of the cases that stayed close to the data [40]. Common threads and differences were searched and identified across the interview data set [42]. Program documents were analyzed using a qualitative descriptive approach using the documents as an additional perspective on the services. Data were organized, initially reviewed to determine what data were available to analyze (superficial examination), read through (thorough examination), coded initially using the study objectives as a guiding framework, and then inductively to draw other insights from the document. These data were then interpreted as part of the case study dataset as a whole [43].

## Results

### Program Characteristics

Thirty participants (seven program staff, 15 program volunteers, and eight clients) participated, with program staff and volunteers attending focus groups and clients participating in interviews.

The included programs aimed to support people in regaining the confidence and skills required to live independently and remain socially engaged in their community through peer support, practical assistance and signposting to other programs. Each program supported older adults (55 or older) transitioning home from the hospital post-discharge and recruited volunteers from their respective communities to support the clients.

The programs were designated as time-limited services, lasting up to eight weeks from time of discharge from hospital. Services were provided within the clients’ homes and communities. Although clients were not required to travel to an office or community setting to participate in the program, many were accompanied by volunteers on shopping excursions or received transportation to and from medical appointments. Each intervention was tailored to the goals and needs of the clients to support their return to independence and social participation. Please see Table 2 for further details.

**Table 2:** Program characteristics of each case.


PROGRAM	*UK PROGRAM 1: HOME FROM HOSPITAL SERVICE*	*UK PROGRAM 2: HOME FROM HOSPITAL SERVICE*

**Program Purpose**	Help older adults as they transition from the hospital to home and regain independence	Provide older adults transitioning home from hospital services

**Staffing Model**	2 full-time dedicated staff; approximately 70 volunteers	3 staff (full and part time); approximately 50 volunteers

**Program Clientele and Admission Criteria**	People over 55 years of age who live alone, and are unable to live independently in the short term	People over 75 years of age who live alone or with a carer have limited or no social support and no social care package

**Services Provided**	8-week service provided. Needs assessment conducted. Support included: shopping, light housework, aid to attend healthcare appointments, collecting prescriptions, emotional support, and signposting to other services.	8-week service. Needs assessment conducted Assistance with meal making, dog walking, gardening, shopping, collecting prescriptions, transportation to appointments, seated exercises, and befriending.


As part of intake, clients underwent risk assessments, including health and well-being assessments at home and environment/safety checks. Table 3 summarizes the risks and conditions assessed by the home from hospital services.

**Table 3:** An overview of the assessment of risks and concerns within the UK programs.


UK PROGRAM 1 (HOME FROM HOSPITAL SERVICE)	UK PROGRAM 2 (HOME FROM HOSPITAL SERVICE)

– Health and safety check sheet includes assessment of outside property, inside property, and other areas as required – Assessment included client’s self-reported considerations of their health (emotional and physical), environment (situation and condition), resources (financial, home, care and travel), background and social relations (family/neighbours/friends supporting- separation and bereavement)	– Referral forms and first contact checklists consider and assess health, wellbeing, safety and security. Specifically, staying well at home, feeling safe at home, managing money well, and staying active, social and healthy – Assessment checklist includes self-reported considerations of health and wellbeing, emotional health, social connectedness, practical support.


Based on the analysis, the study authors identified three themes to describe the facilitators and barriers to program delivery and four themes within the perspectives of program providers and clients.

### Facilitators and Barriers to Program Delivery

Program developers and volunteer coordinators provided insights into the facilitators and barriers to program development and delivery. Three sub-themes were determined i) Relying on short term funding to do a lot ii) Finding and keeping great volunteers, and iii) Integrating into the health and social care systems.

#### Facilitators and barriers to program delivery: Relying on short-term funding to do a lot

Program managers and volunteer coordinators described a range of facilitators and barriers to program design and delivery. Participants consistently identified a) funding and the constraints of being a commissioned service, b) volunteer recruitment and retention, and c) integrating the program into the hospital systems and the broader health system.

Program leaders reported that the scope of their program and services provided were determined by the mandate set out by their funder, which was often local government.

*“The only eligibility that we’ve got to access the service is due to our funders, which is people have got to be over 55, pay their council tax within our district, because they want an investment in their population, and they’ve had to have had a hospital admission. But we are only funded to support them up to 8 weeks after discharge.” (Case #1)*

The staff focused on the positive, noting that a short-term service was not a challenge, stating:

*“The nice thing about the Home from Hospital Service is because of the short-term interim thing about it, we do an awful lot in a short time, and we can support many people because once they’re done, we move on to the next person and the next person.” (Case #1)*

The ability to deliver or scale this type of program is often determined by the availability of funding in other regions to support the service:

*“I think there’s an ambition to roll it out, make it a key service within our larger organization, but it’s going to depend on where funding can be obtained.” (Case #2)*

#### Facilitators and barriers to program delivery: Finding and keeping great volunteers

Although programs had a large pool of volunteers, the episodic nature of the volunteerism could be challenging, with some volunteers going on extended vacations or having their own health issues to manage. Occasionally there weren’t enough volunteers to meet a particular need.

*“And while we support people to do their shopping or doing the shopping for them, depending on their mobility, one of our issues is having enough drivers to deliver that.” (Case #2)*

Or volunteers are not keen on the tasks required by clients:

*“But because of the range from transporting somebody to a hospital appointment to taking them shopping to changing their bed, there’s an awful lot of things that some people might not like to do. So we have to try and match the client with the volunteers.” (Case #1)*

All the volunteer opportunities required a rigorous screening and training process, which was noted to be time-consuming. While some volunteers were ready to start immediately, others required more support. One strategy Case #2 used was a buddy system between the volunteers.

*“Some people, within a couple of hours, can just go and do the job, no problem. Other people need a couple of two-hour sessions and perhaps even a couple of supported sessions to get the confidence to go solo. So, I give them whatever they need individually. Once they’re up and running, I try to buddy them up so if they come up against a problem they’re not sure how to deal with, they’ve got somebody they can contact”*

However, it was noted that only some people who want to volunteer are a good fit for the program.

*“I will assess to see whether they’ve got the right sort of interpersonal skills. Then I usually take them through the sort of things that we’re likely to do, to give them like forewarning of what to expect. Not everyone wants to do this kind of volunteer work or the job centre advised them to volunteer.” (Case #1)*

#### Facilitators and barriers to program delivery: Integrating into the health team and systems

When sharing their experiences of trying to connect with other health services on behalf of a client who needed adaptive equipment, one volunteer coordinator shared the challenge of engaging with the broader health and social system:

*“I spoke to social services, the discharge team, the occupational therapist, and nobody could accept my request. Then, I had to speak to a doctor to get her to make the request. The doctor took the request but couldn’t discuss anything with me. So it was quite frustrating because, at the end of the day, we all have that one person’s best interest at heart. But it’s frustrating.” (Case #1)*

When asked what they thought would happen if their program was unavailable, a program lead from Case 1 suggested that “*Social Services would just be overrun, I think.”*

*“I wouldn’t say we are yet part of the structure, but we are getting recognized… I had a huge leap forward this Tuesday when I saw my name and our organization on the handover notes. And to me, that was almost a supreme accolade.” (Case #2)*

### Perspectives of Program Providers (Staff and Volunteers) and Clients

The study participants provided valuable insights into their experiences with the program and what programmatic factors contributed to supporting independent living and social engagement in the community. Four sub-themes were determined across all the included programs i) *Reducing the burden on family and friends and putting the clients’ minds at ease*, ii) *Valuing psychosocial support*, iii) ‘*Bridging the gap’ between hospital care and independent living* and iv) *Demonstrating the potential for improved client outcomes*.

### Reducing the burden on family and friends and putting the clients’ minds at ease

Many clients stated that they preferred not to rely on friends and family for support as they transitioned from hospital to home. Instead, the clients of the practical transition support programs were more comfortable receiving support through a formalized volunteer service that could help with daily tasks until they could do those activities independently. When a client was asked how she would cope if she did not have a volunteer, she stated:

“*I dread to think I would just have been reliant on friends, and you’d just get more and more embarrassed about what they were doing for you because you can’t keep asking.”* (Client, Case #2).

Moreover, receiving services from a volunteer vetted by a trusted community organization instilled a sense of comfort. One client said, “I just knew they would send you someone you could trust.” The engagement of an established community organization also provided comfort and relief to families and caregivers of clients. She also shared that her daughter felt more at ease knowing that she had help:

*“Oh yes, it’s taken a lot off her mind knowing that I am being looked after somewhere along the line. If it’s not the district nurses, I’ve got <organization name> people. You know, somebody coming in to look after the sanitary things. So it’s taken a lot off her mind. She’s got enough problems of her own.” (Client, Case #1)*

The program was seen to serve as a ‘safety net’ for the clients,

*“I needed somebody today to go and be available for an equipment delivery. They will not deliver equipment without somebody being on the premises. I contacted the office, and the office phoned around for me to find a volunteer to cover for that to take place. Otherwise, that person had nobody to let anyone in. And the hospital doesn’t have the staff.” (Volunteer Coordinator, Case #2)*

Sometimes the staff and volunteers uncover needs that were not identified during the hospital stay:

*“And we often come across things that in hospital might not have been as apparent, but when you visit somebody in their own home, you can quite quickly realize that they’ve got memory issues or memory problem.” (Program Volunteer, Case #1)*

*“But because the volunteer is going in once a week, they will pick up on things, and that feedback then enables us to signpost them on to get the proper help that they need.” (Program Coordinator, Case #2)*

Sometimes clients share things with the TSO staff/volunteers that they would not have with clinicians:

“*Because we’re a voluntary service, sometimes the service user opens more when we go out to see them because it’s not so official as in hospital and social care is involved. Sometimes they hold back about what’s going on because they’re worried that they’d have to go into a home.” (Program Coordinator, Case #1)*

*“We’ve got a checklist for health, environment, relationships, background, and support. Is the family supporting them? What’s their health generally like? They might have been in hospital for six weeks, but that doesn’t mean that they’ve received support for their hearing problems or anything like that. So, we can then refer them on to get support with that.” (Program Coordinator, Case #1)*

In addition to completing formal safety assessments, the staff and volunteers have discovered techniques to check in on clients’ ability to manage at home. For example, one program delivers a ‘welcome home’ basket of tea, biscuits, soup and other necessities upon discharge. This provides an opportunity to see more of the house:

*“I always ask, ‘Would you like me to put the hamper in the kitchen?’ And 99% of the time, they say yes because it’s quite heavy. And you can tell a lot by a person from the kitchen. I look at everything. It’s not just about talking to them, it’s like you’re assessing every little thing.” (Program Coordinator, Case 1)*.

### Valuing psychosocial support

Clients often expressed loneliness and anxiety about returning home from the hospital. The lack of social support or friends and family living a considerable distance away or unavailable for daily or weekly visits increased their worry. As a result, social support or the concept of ‘befriending’ was identified as a valuable component of the programs.

“*The volunteer comes around once a week and helps me sweep up and other odd things. But the nicest part is the chat we have over a cup of tea. She has time to talk, and I always feel better when she has been here.”* (Client, Case# 1)

In addition, the program coordinators described the concept of friendly visiting (‘befriending’) as a program foundation, building a relationship with the client and helping develop their confidence to physically move about their homes and engage in instrumental activities of daily living.

### ‘Bridging the gap’ between hospital care and independent living

The staff and volunteers described the programs as filling the gap between the hospital, home and community. Program volunteers discussed how they help ensure that support is re-established when individuals return home. In that sense, they viewed themselves as more than volunteers, they were carers:

“*And I think our role essentially is that… we’re carers at the moment. There are many different aspects, and they’ve all been fragmented, so I am filling the gap. I’m forever trying to liaison on their behalf to GPs and nurses*.” (Volunteer, Case #1)

In addition to befriending, the programs included a formal signposting or referral process to other community resources and services based on client needs for longer-term services in the community.

### Demonstrating the potential for improved client outcomes

Many coordinators believe hospital discharge could be expedited or hospital readmissions could be reduced through these transition support programs. Program coordinators considered how volunteers’ various supportive tasks could be linked to improved outcomes. While program developers believed that client outcomes could be improved with their services, they also noted that these outcomes needed to be systematically and consistently measured and reported. Demonstrating impact is particularly important to secure funding from health authorities, donors and other sources. One Home from Hospital coordinator noted:

“*Reducing social isolation has an impact on readmission to hospital. So does reducing dropped medical appointments and picking up on things that have been interfered with by admission to hospital. We must ask about it and measure it*.” (Program Coordinator, Case #2)

## Discussion

This study describes two programs delivered by UK-based TSOs designed to support older adults transitioning from hospital to home and returning to independent living. The programs provided a range of personalized support for older adults through services focused on instrumental activities of daily living and psychosocial supports, which address many of the post-discharge needs well documented in the literature [44,45,46,47], including well-being assessments, support for instrumental activities of daily living, psychosocial support, and individualized services directed by the needs and preferences of the service user. The programs were not commissioned to provide any personal care support (hygiene, medications, etc.), and these activities were out of the scope of the service. However, the program managers, coordinators, volunteers, and clients strongly endorsed the value of these types of programs in helping them return to independence at home. Therefore, these results provide an opportunity to discuss the role of TSOs in transitional care, bridging the gap between hospital and home and/or community and the benefits to older adults, their families, and the health care systems.

Intersecting health and social issues often complicate the transition process and adversely affect physical recovery, mood, social participation, quality of life and ultimately, the self-management and self-care capacity of patients and caregivers [29,48,49,50]. Clients benefited from various instrumental activity supports, such as transportation, grocery shopping, and light housekeeping. Clients also appreciated the psychosocial support provided by program volunteers, which aligns with other literature on patients being reluctant to request (more) support from their social networks [51]. A key finding of our research was the value older adults placed on not relying on family and friends to meet these needs and the perception that this benefited their family members. Family and friends often support older adults transitioning from one care setting to another and living at home [52,53]. It is also understood that this informal caregiving is stressful and impacts the caregivers’ health and well-being [54,55,56,57]. Although this was not the focus of this study, the value of transitional care interventions delivered by TSOs for family caregivers warrants further exploration.

The focus on psychosocial support within the programs included in this study and the high value placed on this aspect of the program by study participants align with current literature on the importance of social support [58,59]. Importantly, participants from one program identified the program’s effect on reducing social isolation and, consequently, hospital readmissions. Social isolation and loneliness are associated with poor physical and mental health outcomes, including higher rates of depression and cognitive decline [60]. This is of particular concern among community-living older adults, with a prevalence of social isolation and loneliness as high as 34.0% [61]. Social isolation is also a significant risk factor for older adults following hospitalization [62], and evidence suggests that volunteers can provide this social support as part of a multifaceted program for community-dwelling older adults [63].

The transitional support services of our included TSOs tend to focus on psychosocial support and community participation. As health systems worldwide tackle social isolation and re-structuring healthcare to improve health and social care integration [64], the voluntary sector may become an invaluable partner that can provide services not necessarily provided by health system partners. Transitional support services in the health system do not typically address social problems directly. Still, they refer clients to TSOs, such as those included in this study, for activities focused on community engagement and participation [65,66]. Rather than expanding existing transitional care programs to address these needs or leaving these needs unaddressed, established TSO programs and professional staff can collaborate with a targeted focus on needs related to functional ability and independence in the home. TSO voluntary staff are also optimally positioned to provide support with instrumental activities of daily living and facilitate social participation outside the home.

The participation of TSOs in the provision of health services has been widely discussed [67,68,69,70]. The engagement of these agencies has often been instigated as a response to health system reforms or constrained resources, with health system leaders turning to partnerships to support improved patient care [71] or to expand the breadth and quality of health and social services [72,73,74,75,76,77]. Internationally, advocates call for health and social partnerships to address poorly coordinated and fragmented care [78], concentrating on hospital discharge among older adults to support post-discharge service delivery [79]. There is increasing interest in leveraging the voluntary sector within intermediate care or services that offer time-limited services supporting continuity and quality of care at the interface of hospital, home, long-term care, primary care and community services [33].

Notably, in our study, volunteers did not provide personal care support as these activities were outside the programmatic scope. In the context of strained health system resources, however, this study contributes evidence that TSOs and their volunteers can provide many of the non-clinical aspects of care essential to successful hospital-to-home transitions consisting of psychosocial-coordinative support, physical-cognitive activation, and assistance with medication administration [80]. Volunteer-based interventions can effectively extend the reach of public sector-funded services and provide programs and services most responsive to the needs of their community members. Our study findings also indicated that each program drew volunteers from their respective communities, which aligns with the volunteer literature. Volunteers within TSOs are most often from the communities they serve and thus possess a unique understanding of the needs of their community members as well as community resources [69,77,81]. Additionally, with knowledge of the available community assets, volunteers are well-positioned to match older adults to community resources required to live independently and with meaning [82]. Volunteers in our study also provided peer support grounded in their own lived experiences. The benefits of peer-client relationships based upon shared experiences have been found to validate the recipient [32] and instrumental and information support [83].

## Strengths and limitations

This study has several strengths. The multi-perspective approach to data collection provided a robust triadic—staff, volunteer and client—understanding of voluntary service delivery, which is often absent from the care transition evidence base. Similarly, our study of two programs, including thirty participants, provided rich information triangulated with individual and organizational documents. This study focused on context-bound services and jurisdictions. Although generalizability is not considered the objective of qualitative research, authors recognize that findings from the naturalistic case studies may be challenging to transfer to other contexts. Furthermore, Despite TSOs’ engagement in the provision of health services, they only represent a small portion of service delivery, even in countries with robust voluntary sector engagement in health systems [84,85]. It is important to note that the engagement of TSOs, and the services they provide within their communities highly depend on the broader socio-political environment they are situated within, and study results may not be directly transferrable. Thus, existing literature must be considered within the socio-political context in which it was generated. This study’s topic is framed within a UK conceptualization of third-sector engagement in health service delivery.

## Conclusion

This study provides an overview and discussion of two transition support programs delivered by TSOs. Through the analysis and discussion of these types of services, we have highlighted facilitators and barriers and the value of these programs for older adults transitioning from hospital to home and returning to independent living. The findings suggest that the programs can provide patients with a ‘safety net’ and promote independent living. Having skilled volunteers can positively impact older adults’ experience returning home. When the programs under study are considered in tandem with existing evidence, it facilitates a discussion of how TSO services could be made available more widely to support older adults in their transition experiences.
